# Supplementary material for: Assessing the impact of human error factors on railway accident severity: Evidence from accident investigation reports in Korea
Source: PLoS One. 2026 Mar 27;21(3):e0345753. doi: 10.1371/journal.pone.0345753 (PMC13028362; doi:10.1371/journal.pone.0345753)
Supplement: S1 File — (DOCX) [file pone.0345753.s001.docx]

# Appendix A. Coding Scheme for Identifying Human Error Factors from ARAIB Railway Accident Investigation Reports

***A.1. Introduction***

This appendix provides the detailed coding scheme (codebook) used to extract the eight human error categories (C1–C8) from the railway accident investigation reports issued by the Aviation and Railway Accident Investigation Board of the Republic of Korea. Each category is defined in operational terms, accompanied by explicit coding rules and illustrative excerpts from the investigation reports. The purpose of this codebook is to ensure transparency and reproducibility of the data construction process.

***A.2. General Coding Principles***

The coding follows several general principles.

First, human error factors are identified exclusively from Section 3.2 (“Causes of the Accident”) of each investigation report, and more specifically from the contributory factors described therein. No information outside this section is used for coding.

Second, each contributory factor (whether identified as a primary cause or a contributory cause) is mapped to one and only one human error category (C1–C8). A single causal statement is not allowed to be assigned to multiple categories.

Third, if two or more contributory factors in the same report correspond to the same category, that category is still coded as 1, because all categories are represented as binary indicators.

Fourth, the categories are not mutually exclusive; multiple categories may therefore be assigned to the same accident if different contributory factors correspond to different categories.

Fifth, all coding decisions are based strictly on the textual content of the investigation report. No inference, speculation, or external information is used beyond what is explicitly stated in the report.

Sixth, during the independent coding rounds, each coder performs the coding process individually without consulting or discussing the cases with the other coder, in order to preserve the independence of the initial coding judgments.

Finally, if the applicability of a category remains ambiguous after the independent coding rounds, the case is flagged and resolved through discussion and expert consultation, as described in the Methodology section.

***A.2. Category Definitions, Coding Rules, and Examples***

C1. Deficiencies in Supervisory and Managerial Control of Safety-Critical Activities

| **Operational Definition** This category captures failures in supervision, management, or organizational control related to safety-critical activities, including inadequate oversight, missing or ineffective supervisory actions, and failures in enforcing safety responsibilities.  **Coding Rule (Assign C1 = 1 if …)** Assign C1 = 1 if the report explicitly attributes a contributory factor to inadequate supervision, deficient management control, unclear supervisory responsibility, or failure to enforce or monitor safety-related duties.  **Example Excerpts**   - “The station operations manager and the dispatcher handled train entry without complying with operating regulations, resulting in derailment.” - “The work supervisor failed to control the entry of heavy equipment into the track.” - “The operating organization failed to adequately manage staff and enforce safety responsibilities.” |
| --- |

C2. Inadequate Organizational Training and Competency Development

| **Operational Definition** This category refers to deficiencies in training, education, qualification management, or competency development of personnel involved in railway operations or maintenance.  **Coding Rule (Assign C2 = 1 if …)** Assign C2 = 1 if the report states that insufficient training, inadequate education, lack of qualification management, or poor competency development contributed to the accident.  **Example Excerpts**   - “Insufficient training on operational rules and driving regulations.” - “The equipment operator did not sufficiently understand safety-related operating rules.” - “Training on maintenance procedures was not adequately provided.” |
| --- |

C3. Deficiencies in Maintenance, Inspection, or Technical System Integrity

| **Operational Definition** This category captures failures in maintenance, inspection, technical checking, or integrity management of infrastructure, rolling stock, or safety-critical components.  **Coding Rule (Assign C3 = 1 if …)** Assign C3 = 1 if the report attributes the accident to inadequate maintenance, insufficient inspection, missed defects, or failure to ensure the technical integrity of equipment or infrastructure.  **Example Excerpts**   - “Bearing defects were not detected at an early stage.” - “Surface defects of the tongue rail were repeatedly found but no grinding or replacement was performed.” - “The axle bearing maintenance and inspection system was inadequate.” |
| --- |

C4. Inadequacies in Equipment, Emergency Systems, or Human–System Interfaces

| **Operational Definition** This category refers to design flaws, structural weaknesses, or functional inadequacies of equipment, infrastructure, emergency systems, or human–machine interfaces that increase accident risk.  **Coding Rule (Assign C4 = 1 if …)** Assign C4 = 1 if the report identifies structural, design, or functional inadequacies of technical systems, infrastructure, or safety equipment as a contributory factor.  **Example Excerpts**   - “The transition curve was constructed shorter than the design standard due to topographical constraints.” - “The turnout structure was vulnerable to fatigue due to its geometric configuration.” - “The track structure had inherent weaknesses that increased stress on the rail.” |
| --- |

C5. Noncompliance With or Circumvention of Safety Regulations and Procedures

| **Operational Definition** This category captures violations, noncompliance, or circumvention of established safety rules, procedures, or operational regulations.  **Coding Rule (Assign C5 = 1 if …)** Assign C5 = 1 if the report explicitly states that safety regulations, procedures, or operational rules were violated, ignored, or bypassed.  **Example Excerpts**   - “The train was operated with a load exceeding the allowable limit.” - “The controller did not apply the emergency response manual.” - “Safety procedures for securing cargo were not followed.” |
| --- |

C6. Failures in Communication, Coordination, or Information Transfer

| **Operational Definition** This category refers to failures in communication, information sharing, coordination, or transmission of safety-critical information between personnel, departments, or systems.  **Coding Rule (Assign C6 = 1 if …)** Assign C6 = 1 if the report attributes the accident to miscommunication, missing information transfer, incorrect information, or lack of coordination.  **Example Excerpts**   - “The dispatcher provided incorrect speed information.” - “Relevant design changes were not communicated to the maintenance department.” - “Critical operational information was not shared among staff.” |
| --- |

C7. Deficient Operational Decision-Making or Task-Related Competence

| **Operational Definition** This category captures poor judgment, incorrect operational decisions, or insufficient task-related competence in performing safety-critical actions.  **Coding Rule (Assign C7 = 1 if …)** Assign C7 = 1 if the report states that incorrect judgment, poor decision-making, or lack of task competence directly contributed to the accident.  **Example Excerpts**   - “After the initial accident, the local controller failed to display a stop signal.” - “The operator conducted shunting operations despite unresolved route conflicts.” - “The staff member failed to make an appropriate safety-related decision.” |
| --- |

C8. Performance Degradation Due to Fatigue or Physical and Health Conditions

| **Operational Definition** This category refers to performance degradation caused by fatigue, drowsiness, or other physical or physiological conditions affecting human performance.  **Coding Rule (Assign C8 = 1 if …)** Assign C8 = 1 if the report explicitly mentions fatigue, drowsiness, health conditions, or reduced alertness as a contributory factor.  **Example Excerpts**   - “The train driver ignored the stop signal due to drowsiness and reduced alertness.” - “Fatigue caused by inadequate rest management affected the driver’s performance.” |
| --- |
