# Supplementary material for: Assessing the impact of human error factors on railway accident severity: Evidence from accident investigation reports in Korea
Source: PLoS One. 2026 Mar 27;21(3):e0345753. doi: 10.1371/journal.pone.0345753 (PMC13028362; doi:10.1371/journal.pone.0345753)
Supplement: Attachment — Submitted filename: PLOS_ONE_Review_Report.pdf [file pone.0345753.s002.pdf]

Manuscript information

Manuscript title: "Assessing Human Error Contributions to Railway Accident Causation: Evidence from Accident Investigation Reports in Korea"

Recommendation: Minor revision.

Competing interests statement: I declare no competing interests.

Comments to the Authors

Summary assessment

The manuscript addresses an important problem: identifying and quantifying the determinants of "human error" in railway incidents based on official ARAIB investigation reports. The proposed classification of factors (C1–C8) and the attempt to link these factors to incident costs could constitute a valuable contribution, provided that key definitions are clarified, the case-selection criteria are made internally consistent, and methodological reporting and reproducibility are strengthened.

Below I provide comments that, in my view, are necessary for the manuscript to meet PLOS ONE standards regarding technical rigor, transparency, and reproducibility.

Major comments

1. Dependent variable "accident cost" and temporal comparability

    - Please provide a clear definition of the cost measure (its components, where it is sourced from in the reports, and whether it is an estimate produced by the commission, the operator, or the regulator).

2. Inconsistency with event inclusion criteria (KRW 50 million threshold vs. minimum in the table)

    - The text indicates a material-damage threshold of > KRW 50 million, whereas the descriptive statistics table reports a minimum cost of KRW 0.11 million (and a mean of KRW 46.98 million).
    - Please clarify whether the KRW 50 million threshold applied only to material damage or to a different definition of "cost"; and whether incidents meeting other inclusion criteria (e.g., casualties) were retained despite low material damage.

3. Appropriateness of the model choice and model comparison

- Comparing a log-linear OLS model (costs) with Poisson/NB models (incident counts) is methodologically inconsistent (different dependent variables and scales).
- If the dependent variable is cost, please consider a GLM with a Gamma distribution or a lognormal specification.

4. Control for confounding and risk of omitted-variable bias

- Please assess whether C2/C5/C6/C7 are truly "negligible" or whether their apparent lack of effect is masked by co-occurrence patterns with C1/C3/C4.

5. Coding reliability and reproducibility of the extraction procedure

- Please describe who performed the coding, whether double-coding was used, and report an inter-rater agreement metric (e.g., Cohen's kappa).
- Please provide the coding scheme (codebook) with category definitions and examples of report excerpts that lead to an assignment of "1"; Figure 3 is currently difficult to read/interpret.
- Please indicate how ambiguous cases were handled and resolved.

6. Data and code: PLOS ONE transparency requirements

- Please provide an explicit Data Availability Statement specifying: where exactly the reports and/or extracted materials are located; whether the dataset of 105 observations and the C1–C8 variables can be shared; and, if not, what legal constraints apply and how alternative access can be obtained.

Minor comments

1. Figure numbering and internal consistency

- The manuscript contains inconsistencies, including duplicate numbering of "Figure 1" and "Figure 3" for two different figures. Please harmonize figure numbering, in-text citations, and captions.

2. Definitions of categories C1–C8

- While the classification table is helpful, please add a brief theoretical rationale for each category and provide example phrases/findings from the reports that lead to an assignment of "1".

3. Study limitations

- The limitations section is appropriate, but it should be strengthened by articulating concrete implications for inference (e.g., selection bias: only officially investigated incidents; cost measurement error; binary simplification).

Concrete edits (location + suggested fix)

- **Abstract and Highlights**
  Standardize UK vs US spelling consistently (e.g., *organisational* → *organizational; modelling* → *modeling; prioritising* → *prioritizing* if US is selected).
- **Keywords:** remove trailing punctuation and standardize separators; avoid a final semicolon after the last keyword.
  Ensure the Highlights spelling matches the spelling variant used in the Abstract and main text.
- **Introduction – punctuation and readability**
  Improve sentence structure where very long sentences reduce clarity; add commas where needed.
  Example edit (improves readability and parallelism):
  Original: *"Railway employees including operators signal staff maintenance workers and construction personnel…"*
  Suggested: *"Railway employees—including operators, signal staff, maintenance workers, and construction personnel—…"*
  Add comma after introductory phrase:
  "Under such conditions even minor…" → *"Under such conditions, even minor…"*
  Add commas in lists and after introductory dependent clauses, e.g.:
  "…fatalities and injuries these accidents impose additional burdens such as…" → *"…fatalities and injuries, these accidents impose additional burdens, such as…"*
  Insert a comma before a non-finite clause when it functions as an added consequence:
  "…preventive practice limiting…" → *"…preventive practice, limiting…"*
- **Literature Review – typos and citation consistency**
  "Amstrong, 2021" → *"Armstrong, 2021"*
  "semi structured" → *"semi-structured"*
  Standardize *analyse/analyze* depending on your chosen UK/US variant.
  "Starter & Alexander, 2000" → *"Sarter & Alexander, 2000"* (and ensure consistency with the reference list).

One in-text citation appears without a year (e.g., *"Nawaz & Qayyum)."*); insert the correct year to match the reference list.

- **Methods/Results – terminology and minor editorial points**
  Harmonize figure references (Figure numbering appears inconsistent in places; while not purely a language issue, it affects readability and credibility).
  Standardize *modelling/modeling* (and any other UK/US spellings) throughout.
  Section heading: *"5. Result"* → *"5. Results"* (standard academic convention).
  Align factor names (C1–C8) with the exact wording used in the relevant table for terminological consistency (e.g., "Supervision and Management" vs "Supervisory and Management").
