# Supplementary material for: Assessing the impact of human error factors on railway accident severity: Evidence from accident investigation reports in Korea
Source: PLoS One. 2026 Mar 27;21(3):e0345753. doi: 10.1371/journal.pone.0345753 (PMC13028362; doi:10.1371/journal.pone.0345753)
Supplement: Attachment — Submitted filename: Response_to_Reviewers.docx [file pone.0345753.s004.docx]

**Response to Reviewers**

**Manuscript Number:** PONE-D-25-51103
**Title:** Assessing the impact of human error factors on railway accident severity: Evidence from accident investigation reports in Korea

**Authors:** Changhun Kim, Jun Lee*

**General Response**

We sincerely thank the Academic Editor and the reviewers for their careful reading of our manuscript and for their constructive and insightful comments. We believe that these suggestions have substantially improved the clarity, rigor, and transparency of our study.

In response to the comments, we have:

- Revised the title, abstract, and framing to focus explicitly on accident severity rather than causation.
- Adopted a Gamma GLM as the main model, with log-linear OLS, Ridge, and Lasso used as robustness checks.
- Added systematic model comparisons, robustness checks, and cross-validation results.
- Substantially strengthened the methodological framework, especially the coding procedure.
- Added inter-coder reliability analysis (Cohen’s kappa).
- Expanded and strengthened the limitations section.
- Added concrete examples of how investigation report text was mapped to coding categories (C1–C8).
- Updated the Data Availability statement and deposited the dataset and code in a public repository.
- Corrected and unified the funding disclosure according to PLOS ONE policy.
- Carefully proofread and corrected the reference list and language throughout the manuscript.

All changes in the manuscript are highlighted in the tracked-changes version.

Below, we provide a detailed, point-by-point response to each comment.

**Response to the Academic Editor / Journal Requirements**

We sincerely thank the Academic Editor, Dr. Qing-Chang Lu, for the careful editorial assessment of our manuscript and for the detailed and constructive guidance regarding the journal’s requirements. We are grateful for the opportunity to revise our manuscript and have carefully addressed all editorial comments concerning formatting, data and code availability, and funding disclosure. We believe that these revisions have substantially improved the transparency, reproducibility, and compliance of the manuscript with PLOS ONE’s policies. Our detailed, point-by-point responses to each editorial requirement are provided below.

**Comment 1: PLOS ONE Style & File Naming Requirements**

Please ensure that your manuscript meets PLOS ONE's style requirements, including those for file naming.

**Response**

We have carefully revised the manuscript to fully comply with the PLOS ONE style requirements, using the official PLOS ONE templates for the main text and the title/authors/affiliations pages. We have also ensured that all files are named according to PLOS ONE’s file-naming conventions. The revised submission includes (i) a clean manuscript, (ii) a marked-up version with tracked changes, and (iii) the Response to Reviewers file, all formatted according to the journal guidelines.

**Comment 2-4: Code sharing, Data repository, Data availability timing & open data policy**

(Combined) PLOS ONE requires that all author-generated code be made publicly available without restriction, and that the minimal dataset necessary to reproduce the findings be deposited in a stable, public repository. In addition, authors are encouraged to finalize their data sharing plan at the revision stage rather than upon acceptance.

**Response**

We thank the editor for these important clarifications and have fully revised our data and code sharing strategy to comply with PLOS ONE’s open science and reproducibility policies.

We have now deposited (i) the minimal anonymized dataset required to reproduce all results and (ii) the complete set of author-generated analysis code in a stable public repository (Zenodo). The repository contains all scripts used for data processing, variable construction, statistical modeling, and figure generation, and is sufficient to fully reproduce all analyses reported in the manuscript: https://doi.org/10.5281/zenodo.18310486

The primary sources of this study are the official railway accident investigation reports published by the Aviation and Railway Accident Investigation Board (ARAIB) of the Republic of Korea, which are publicly available in Korean via the ARAIB website. However, due to institutional and copyright-related restrictions, we are not permitted to redistribute the full set of original investigation reports as a compiled dataset. Instead, we provide a fully structured and anonymized dataset consisting of 105 observations (including the constructed C1–C8 variables and accident cost), together with the complete analysis code.

We have updated the Data Availability Statement to clearly explain:

(i) the origin of the source documents,

(ii) why the original reports cannot be redistributed,

(iii) what exact data and code are publicly provided, and

(iv) how readers can directly access the original reports via the ARAIB website.

Finally, we have revised our data sharing plan so that all data and code are already openly available at the revision stage, and no access restrictions will apply upon acceptance. The manuscript now fully complies with PLOS ONE’s open data and code sharing policies.

**Comment 5-6: Funding disclosure & Acknowledgments inconsistency**

There is an inconsistency between the Funding Statement and the Acknowledgments section. Funding information should not appear in the manuscript body and must be declared consistently in the Funding Statement of the submission system.

**Response**

We thank the editor for pointing out this important inconsistency. We have now corrected and unified the funding disclosure in full compliance with PLOS ONE’s policy. Specifically, we have removed all funding-related text from the Acknowledgments section and from the manuscript body. The funding information is now declared only in the Funding Statement of the submission system, as required by PLOS ONE.

**Comment 7: Reviewer-suggested citations**

If the reviewer comments include a recommendation to cite specific previously published works, please review and evaluate these publications to determine whether they are relevant and should be cited. There is no requirement to cite these works unless the editor has indicated otherwise.

**Response**

We have carefully reviewed all publications suggested by the reviewers and evaluated their relevance to the scope and contribution of the present study. We have cited those works that are directly relevant to our research questions, methodological framework, and interpretation of the results, and have incorporated them into the revised Introduction and Discussion sections. Where the suggested references were not directly relevant to the specific focus of this study or duplicated content already cited, we have not included them, in accordance with the editor’s guidance.

**Response to Reviewer #1**

We sincerely thank Reviewer #1 for the constructive and insightful comments, which have helped us substantially improve the clarity, transparency, and rigor of the manuscript. We have carefully revised the manuscript accordingly. Our detailed responses are provided below.

**Comment 1**

While the introduction effectively identifies a historical gap in the literature (citing sources from 2002, 2009, and 2011), it would be more compelling if the authors could demonstrate that this gap persists today by citing more recent literature.

**Response**

We have revised and expanded the Introduction to incorporate recent literature and to clearly demonstrate that the identified research gap persists in the current literature, while maintaining the original narrative flow and motivation of the study.

**Page 4, Line 3 - Page 4, Line 24** of our paper:

- Added recent references (2022–2024) to the Introduction.
- Rewrote the literature review paragraph(s) in the Introduction.

**Comment 2**

The methodology section mentions that investigation reports were selected based on “incidents of significant safety and operational relevance.” This is too vague. The authors should provide precise inclusion and exclusion criteria.

**Response**

We have revised the data source and sample construction section to explicitly describe the legal scope of reportable accidents and to clearly specify the inclusion and exclusion criteria used to construct the final analytical sample.

**Page 8, Line 24 - Page 9, Line 7 & Page 10, Line 15 - Page 11, Line 2** of our paper:

- Added a description of the legal criteria defining reportable accidents and clarified that satisfying any one criterion is sufficient for an accident to be investigated.
- Added an explicit description of the sample construction process, including the total number of reports reviewed, the final sample size, and the exclusion criteria.

**Comment 3**

The paper describes a “structured extraction and coding procedure” but provides no details on its implementation.

**Response**

We have expanded the coding methodology section to document the coding rules, reliability checks, and the full coding workflow.

**Page 12, Line 7 - Page 14, Line 18** of our paper:

- Added a table specifying the operational definitions and representative report-based evidence for categories C1–C8.
- Added a description of the binary coding rules and decision criteria.
- Added inter-rater reliability results (Cohen’s kappa) and the double-coding procedure.
- Added a description of the disagreement resolution process and expert review.
- Added a figure illustrating the overall coding workflow.

**Comment 4**

The reference list requires careful proofreading.

**Response**

We have reviewed and corrected the entire reference list to improve consistency and formatting.

**Throughout the manuscript:**

- Reformatted the reference list to comply with the PLOS ONE reference style.
- Checked and corrected inconsistencies between in-text citations and the reference list.
- Corrected typographical errors and incomplete bibliographic information in the reference list.

**Comment 5**

The snippets suggest the paper’s contribution is a new coding framework. It is unclear if the paper goes on to apply this framework and present findings from the analysis.

**Response**

We have revised the manuscript to make it explicit that the proposed coding framework is applied to the full dataset and directly feeds into the empirical analysis.

**Page 14, Line 19 - Page 15, Line 3** of our paper:

- Added and revised Figure 3 to illustrate the full coding workflow from report text to coded variables and analysis-ready dataset.
- Added a description of the end-to-end process linking report review, category assignment, binary coding, and database construction.
- Revised the coding procedure section to explicitly describe how the coded variables are used as inputs to the statistical analysis.

**Response to Reviewer #2**

We sincerely thank Reviewer #2 for the thorough and insightful review and for the detailed comments on the conceptual framing, statistical methodology, and interpretation of the results. We have carefully revised the manuscript in response to these comments to improve conceptual clarity, strengthen the statistical rigor of the analysis, and clarify the limitations and scope of interpretation. In particular, we have revised the framing to focus explicitly on accident severity, added additional model diagnostics and robustness checks, and expanded the discussion of limitations. Our detailed, point-by-point responses are provided below.

**Comment 1**

Model assumptions are not validated; no diagnostics are provided. Model explanatory power is weak (adjusted R² ≈ 0.08) and requires explicit discussion.

**Response**

We have revised both the methodological framework and the interpretation of the results to address the concerns regarding model validity and limited explanatory power. Instead of relying on OLS assumptions, we adopted Gamma GLM as the baseline specification and added systematic model comparison and robustness checks. In addition, we expanded the Discussion section to explicitly acknowledge and discuss the limited explanatory power and data-related constraints.

**Page 19, Line 14 - Page 22, Line 17** & **Page 26, Line 9 - Page 27, Line 21** of our paper:

- Revised the modeling framework to adopt a Gamma GLM with a log link as the baseline specification instead of relying on log-linear OLS.
- Added a formal comparison between the Gamma GLM and the log-linear OLS model using log-likelihood, AIC, and deviance.
- Added predictive performance comparisons using RMSE and MAE.
- Added robustness checks using OLS, Ridge, and Lasso with cross-validation.
- Expanded the Discussion section to explicitly discuss limited explanatory power, selection effects, measurement noise, and limitations arising from binary coding and institutional data constraints.

**Comment 2**

The statistical analysis requires further rigor, including clearer model validation, robustness checks, and clarification of alternative specifications and coding procedures.

**Response**

The statistical analysis requires further rigor, including clearer model validation, robustness checks, and clarification of alternative specifications and coding procedures.

**Page 19, Line 14 - Page 21, Line 16** of our paper:

- Revised the baseline modeling strategy to adopt a Gamma GLM appropriate for the accident cost data.
- Added a formal comparison between the Gamma GLM and the log-linear OLS model using likelihood-based measures and predictive error metrics.
- Added robustness checks using OLS, Ridge, and Lasso with cross-validation.
  Clarified the scope of alternative model comparisons by focusing on distributionally appropriate specifications.
- Added detailed coding rules and inter-coder reliability results (Cohen’s kappa).

**Comment 3**

The manuscript should focus on accident severity (cost) rather than causation, and causal language should be avoided throughout.

**Response**

We have revised the title, abstract, and the overall framing of the manuscript to clearly reflect that the study analyzes accident severity measured in economic terms, rather than accident causation, and we have removed or revised causal language throughout the manuscript.

**Throughout the manuscript:**

- Revised the title to explicitly refer to accident severity rather than causation.
- Revised the abstract to clarify that the dependent variable is accident cost and that the analysis examines associations with severity.
- Revised the Introduction to frame the study in terms of severity impacts in economic terms rather than causal mechanisms.
- Revised the Discussion and Conclusion to consistently interpret the results as associations with severity rather than causal effects.
- Replaced causal wording (e.g., “determinants,” “drivers,” “causes”) with association-based language throughout the manuscript.

**Comment 4**

The authors should expand the limitations section, clarify the scope and constraints of the data and coding scheme, provide concrete coding examples, consider additional covariates in future work, and avoid causal interpretation.

**Response**

We have expanded the Discussion and Conclusion to explicitly acknowledge the main data and methodological limitations, clarified the scope and constraints of the coding scheme, and revised the interpretation to avoid causal claims.

**Page 26, Line 9 - Page 27, Line 15** of our paper:

- Added an explicit discussion that the dataset is limited to officially investigated serious accidents and does not represent the full population of all railway incidents.
- Added a discussion of potential measurement noise and investigator judgment in the estimation of accident costs.
- Added a clear statement on the limitations of binary coding and the inability to capture the intensity or degree of contributory factors.
- Added repeated cautions that the estimated relationships should be interpreted as associations rather than causal effects.
- Added concrete explanations of the coding procedure and representative examples (Table 2), together with inter-coder reliability results (Table 3), and a full coding scheme with category definitions and illustrative examples in Appendix A.
- Added a statement that future studies should incorporate additional contextual and operational covariates and broader datasets.

**Comment 5**

We have corrected the inconsistency in the funding disclosure and aligned the Funding Statement and the manuscript to comply with PLOS ONE’s funding disclosure requirements.

**Response**

We have removed the funding information from the Acknowledgements and revised the Funding Statement to consistently report the KAIA grant (RS-2023-00239464), clarifying that the funder had no role in the study and ensuring consistency between the manuscript and the submission metadata.

**Comment 6: Overall Evaluation**

The manuscript is promising, but requires stronger statistical validation, expanded limitations, and clearer coding procedures.

**Response**

We thank the reviewer for the constructive overall assessment. In response, we have substantially revised the manuscript to strengthen the statistical validation, expand and clarify the discussion of limitations, and improve the transparency and rigor of the coding procedure.

Specifically, we have revised the modeling strategy by adopting a Gamma GLM appropriate for the accident cost data, added systematic model comparisons and robustness checks, expanded the Discussion to explicitly acknowledge data and methodological limitations, and clarified the coding scheme through detailed explanations, representative examples, inter-coder reliability analysis, and a full coding scheme provided in Appendix A. We believe that these revisions have substantially improved the methodological rigor, transparency, and overall contribution of the manuscript.

**Response to Reviewer #3**

We sincerely thank Reviewer #1 for the constructive and insightful comments, which have helped us substantially improve the clarity, transparency, and rigor of the manuscript. We have carefully revised the manuscript accordingly. Our detailed responses are provided below.

**Major Comment 1**

Please provide a clear definition of the cost measure (its components, where it is sourced from in the reports, and whether it is an estimate produced by the commission, the operator, or the regulator).

**Response**

We clarified the definition, components, source, and institutional origin of the accident cost variable and added an explicit explanation of its temporal comparability.

**Page 9, Line 20 - Page 10, Line 9** of our paper:

- The accident cost variable is now explicitly defined as the sum of property damage and other damage reported in Section 1.2 (Damage Information) of each official investigation report.
- It is now clearly stated that casualties are not monetized and therefore are not included in the cost measure.
- The components of property damage (track, rolling stock, signaling systems, structures and facilities) and other damage (compensation and service disruption–related losses) are explicitly described.
- It is now explicitly stated that all cost figures are official damage assessments finalized and published by the Investigation Board, not estimates from operators or secondary sources.
- An explicit statement has been added that the same legal framework, reporting format, and assessment procedures have been applied consistently over time, providing temporal comparability of the cost measure across years.

**Major Comment 2**

Inconsistency with event inclusion criteria (KRW 50 million threshold vs. minimum cost in table).

**Response**

We clarified that the KRW 50 million threshold applies only to property damage and that accidents meeting other statutory criteria (e.g., casualties) are included even if the reported cost is low.

**Page 8, Line 24 - Page 10, Line 9** of our paper:

- Clarified that an accident is investigated if **any one** statutory criterion is satisfied.
- Clarified that the **KRW 50 million threshold applies only to property damage**, not to total accident cost.
- Clarified that cases with casualties or other qualifying conditions are **included even when the cost is small**, explaining the low minimum value in the table.

**Major Comment 3**

Appropriateness of the model choice and model comparison

**Response**

We revised the modeling framework to use a Gamma GLM appropriate for a continuous, positive, and right-skewed cost variable and removed the inappropriate comparison with count-data models.

**In the Methodology / Model Performance Comparison section:**

- Revised the baseline model to a **Gamma GLM with a log link** for accident cost in levels.
- Added an explicit comparison between the **Gamma GLM and a log-linear OLS model** to assess distributional suitability and numerical stability.
- Restricted alternative specifications and robustness checks to **cost-based models** (log-linear OLS, Ridge, and Lasso), avoiding any comparison with count-data models.
- Reframed the model comparison to focus on **distributionally appropriate specifications for a continuous cost variable**.

**Major Comment 4**

Control for confounding and omitted-variable bias

**Response**

We clarified the interpretation of the non-significant coefficients and added robustness checks to assess whether their apparent lack of effect reflects confounding or co-occurrence patterns rather than true irrelevance.

**In the Results and Discussion sections:**

- Clarified that non-significant coefficients (C2, C5, C7, etc.) should not be interpreted as evidence of no relevance, but as conditional associations given the presence of other factors.
- Added an explicit discussion that several categories frequently co-occur and that the estimated coefficients represent partial associations after controlling for other categories.
- Reinforced the interpretation by referring to the robustness checks using alternative specifications (log-linear OLS, Ridge, Lasso), which show similar patterns in relative importance.
- Added a limitation statement that potential confounding and omitted-variable bias cannot be fully ruled out given the structure of report-based coding and the limited set of covariates.

**Major Comment 5**

Coding reliability and reproducibility of the extraction procedure

**Response**

We substantially strengthened the description and documentation of the coding procedure by adding double-coding, inter-rater reliability statistics, a full codebook with examples, a clearer workflow figure, and an explicit protocol for resolving ambiguous cases.

**In Section 3.3 Coding Procedure and Reliability, Appendix A:**

- Clarified that all cases were **independently double-coded by two researchers**, and reported **inter-rater agreement using Cohen’s kappa** (Table 3).
- Added **representative coding examples** and explicit operational definitions for each category (Table 2), and provided the **full coding scheme in Appendix A**.
- Revised and clarified **Figure 3** to more clearly illustrate the step-by-step extraction and coding workflow.
- Added an explicit description of how **ambiguous cases were discussed, reviewed with an external expert, and resolved by consensus**.

**Major Comment 6**

Data and code availability / transparency

**Response**

We added a detailed Data Availability Statement specifying the location of the original reports, the public repository for the extracted dataset and code, and the legal constraints on redistributing the full investigation reports.

**In the Data Availability section:**

- Specified that the original ARAIB investigation reports are publicly available via the official ARAIB website.
- Clarified that, due to institutional and copyright restrictions, the full set of original reports cannot be redistributed by the authors.
- Stated that the extracted and anonymized dataset (105 observations with C1–C8 and accident cost) and the full analysis code are publicly available in a stable repository (Zenodo), with a DOI (<https://doi.org/10.5281/zenodo.18310486>).
- Explained that these materials are sufficient to fully reproduce all analyses and figures in the paper.
- Added guidance that readers who wish to inspect the original reports can access them directly via the ARAIB website.

**Minor Comment 1**

Figure numbering and internal consistency

**Response**

We corrected all figure numbering inconsistencies and harmonized figure numbers, in-text citations, and captions throughout the manuscript.

**Throughout the manuscript:**

- Corrected duplicate and inconsistent figure numbers.
- Aligned all in-text figure references with the correct figure numbers.
  Updated all figure captions to match the revised numbering.
- Checked the manuscript to ensure global consistency between figures, captions, and references in the text.

**Minor Comment 1**

Figure numbering and internal consistency

**Response**

We corrected all figure numbering inconsistencies and harmonized figure numbers, in-text citations, and captions throughout the manuscript.

**Throughout the manuscript:**

- Corrected duplicate and inconsistent figure numbers.
- Aligned all in-text figure references with the correct figure numbers.
  Updated all figure captions to match the revised numbering.
- Checked the manuscript to ensure global consistency between figures, captions, and references in the text.

**Minor Comment 2**

Definitions of categories C1–C8

**Response**

We corrected all figure numbering inconsistencies and harmonized figure numbers, in-text citations, and captions throughout the manuscript.

**In the Methodology and coding-related materials:**

- Added brief theoretical rationales for each category by explicitly linking C1–C8 to established human factors and organizational safety frameworks (Table 1).
- Added representative phrases and investigation findings used for coding decisions in Table 2.
- Provided the full coding scheme with definitions and illustrative examples in Appendix A.
- Clarified in the text that a category is coded as “1” when at least one corresponding indicator is explicitly identified in the report.

**Editorial consistency, language, and formatting**

We thoroughly revised the manuscript to improve language quality, editorial consistency, spelling and punctuation, citation accuracy, terminology consistency, and overall readability across all sections.

**Throughout the manuscript:**

- Standardized spelling consistently to US English (e.g., organizational, modeling, prioritizing) across the Abstract, Highlights, main text, and keywords.
- Corrected keywords formatting removed trailing punctuation, and harmonized separators and spelling with the main text.
- Revised the Introduction to improve readability by restructuring long sentences, adding missing commas, and standardizing list punctuation.
- Corrected typos and citation inconsistencies in the literature review (e.g., Armstrong, semi-structured, Sarter & Alexander, missing publication years).
- Harmonized figure numbering and in-text references for consistency.
- Standardized terminology and spelling in the Methods and Results sections (e.g., modeling/modeling, section title “Results”).
- Aligned the wording of factor names (C1–C8) between the text and tables to ensure terminological consistency.

**Closing Statement**

We sincerely thank the Academic Editor and all reviewers for their constructive and insightful comments, which have helped us substantially improve the quality, clarity, and rigor of the manuscript. We have carefully revised the paper to address all major and minor points raised in the reviews, including substantial improvements to the methodological framework, data transparency, coding procedure and reproducibility, interpretation and limitations, as well as editorial consistency and presentation.

We believe that these revisions have significantly strengthened the technical soundness, transparency, and overall contribution of the study, and we hope that the revised manuscript now meets the standards of PLOS ONE. We are grateful for the time and effort invested by the reviewers and the editor, and we appreciate the opportunity to resubmit our work for further consideration.
